# Regulation of signal transduction pathways in colorectal cancer: implications for therapeutic resistance

**DOI:** 10.7717/peerj.12338

**Published:** 2021-10-22

**Authors:** Yeelon Yeoh, Teck Yew Low, Nadiah Abu, Pey Yee Lee

**Affiliations:** UKM Medical Molecular Biology Institute (UMBI), Universiti Kebangsaan Malaysia, Kuala Lumpur, Malaysia

**Keywords:** Colorectal cancer, Drug resistance, Chemotherapy, Targeted therapy, Signalling pathways, Mitogen-activated protein kinases, Protein kinase B, Notch receptor

## Abstract

Resistance to anti-cancer treatments is a critical and widespread health issue that has brought serious impacts on lives, the economy and public policies. Mounting research has suggested that a selected spectrum of patients with advanced colorectal cancer (CRC) tend to respond poorly to both chemotherapeutic and targeted therapeutic regimens. Drug resistance in tumours can occur in an intrinsic or acquired manner, rendering cancer cells insensitive to the treatment of anti-cancer therapies. Multiple factors have been associated with drug resistance. The most well-established factors are the emergence of cancer stem cell-like properties and overexpression of ABC transporters that mediate drug efflux. Besides, there is emerging evidence that signalling pathways that modulate cell survival and drug metabolism play major roles in the maintenance of multidrug resistance in CRC. This article reviews drug resistance in CRC as a result of alterations in the MAPK, PI3K/PKB, Wnt/β-catenin and Notch pathways.

## Introduction

Colorectal cancer (CRC) is ranked as the most prevalent malignancy globally, after cancers of the lungs and the breast. In 2020 alone, two million new cases of CRC have been estimated worldwide, whereby 940,000 CRC cases potentially result in mortalities (International Agency for Research on Cancer, 2020). Risk factors of CRC are primarily genetic predisposition and environmental influences. As such, the development of CRC epitomizes gene-environment interaction, and multiple aetiologies that have been ascribed to CRC include genetic disorders (familial adenomatous polyposis and Lynch syndrome), family history of sporadic CRC, as well as unhealthy lifestyle (tobacco smoking, physical inactivity and heavy alcohol consumption) (Stigliano et al., 2014; Macrae, 2016; Yurgelun et al., 2017).

The genetic model of CRC carcinogenesis theorizes CRC as an accumulation of a set of driver mutations occurring in genes essential for the growth and differentiation of intestinal epithelium (Fearon & Vogelstein, 1990). Such mutations dysregulate cell signalling events in the intestinal epithelium, leading to CRC progression. Activation of oncogenes (*e.g.*, KRAS or BRAF) and deletions of tumour suppressor genes (*e.g.*, APC and p53) are known to disrupt cell development which results in uncontrolled cell division and cancer metastasis (Baker et al., 1989; Sansom et al., 2004; Aoki et al., 2007; Raskov et al., 2020).

Individuals diagnosed with CRC are subsequently treated according to the severity of CRC. The treatment options are summarised in Table 1. Surgery is employed to resect early-stage cancer to prevent the metastasis of CRC and its accompanying complications (Rentsch et al., 2016; Dekker et al., 2019). Meanwhile, radiotherapy is often applied as a supplemental treatment before CRC surgery and is targeted towards locally advanced CRC in order to reduce the size of tumour prior to surgery. This renders the surgical procedure less radical and may reduce local relapse (Häfner & Debus, 2016; Ma et al., 2017). On the other hand, systemic treatments of CRC are prescribed when patients suffer from metastatic CRC (mCRC). Systemic treatments can be divided into combination chemotherapy and targeted therapy. In combination chemotherapy, cancer drugs are combined in synergistically to produce more effective cytotoxic effects on the cancer cells (Carethers, 2008; Wolpin & Mayer, 2008). FOLFOX is a standard adjuvant chemotherapy for treating advanced CRC that is made up of folinic acid (leucovorin), oxaliplatin (L-OHP) and 5-fluorouracil (5-FU) (De Gramont et al., 2000). While FOLFOX has been proven effective for treating stage III and IV CRC, FOLFOX might not be suitable for treating high-risk stage II CRC harbouring BRAF V660E mutation with or without microsatellite stable (MSS) status due to a higher chance of tumour relapse after treatment (Seppälä et al., 2015). Clinical trial data also suggests that a 6-month FOLFOX regimen results in significant neurotoxicity for high-risk stage II CRC patients, suggesting the need to reduce the duration of adjuvant chemotherapy for better treatment outcomes (Iveson et al., 2021). XELOX (also known as CAPOX) is an alternative first-line or second-line treatment for high-risk stage II CRC which comprises capecitabine and oxaliplatin. It has been reported that a 3-month XELOX regimen exhibits a similar curative effect to a 6-month FOLFOX regimen. However, treatment benefits vary according to the patients’ medical condition and side effects of the treatment (Guo et al., 2016; Petrelli et al., 2020; Iveson et al., 2021). Targeted therapy on the other hand involves the use of small-molecule drugs or antibodies to specifically target genes or proteins that drive cancer survival and cancer metastasis (Xie, Chen & Fang, 2020a). In most cases, combination chemotherapy and targeted therapy are employed to treat mCRC after surgical removal of tumours (Kuo et al., 2005; Townsley et al., 2006; Berlin et al., 2007).

**Table 1:** List of treatments available for CRC patients.

Treatment method	Characteristic	Reference
Complete mesocolic excision (CME)	Surgery that involves removal of the affected colon and its lateral lymphatic supply by cutting the mesentry.	Dimitriou & Griniatsos (2015)
Single incision laparoscopic surgery (SILS)	Surgery involving the use of one umbilical port.	Greaves & Nicholson (2011)
Natural orifice transluminal endoscopic surgery (NOTES)	Surgery that involves entering the peritoneal cavity via the gastrointestinal tract using a natural orifice.	Kalloo (2007)
Robotics laparoscopic surgery	Minimally invasive bowel resections performed by robotic system.	Giulianotti et al. (2003)
Short-course radiotherapy	Procedure regarding patients being exposed to radiation for 1 week with a clinical dosage of 25 Gray in 5 fractions followed by surgery 1 week later.	Påhlman (1997)
Long-course chemoradiotherapy	Procedure that covers radiation exposure for 5 weeks with a clinical dosage of 45–50 Gray in 25–28 fractions together with 5 concurrent fluoropyrimidine-based regimens as radiation sensitiser. This is followed by surgery 4–8 weeks later.	Bosset et al. (2006)
FOLFOX	Chemotherapeutic regimen involving the use of folinic acid, 5-fluorouracil (5-FU) and oxaliplatin to promote DNA cross-linking, hence inhibiting DNA synthesis and eventually induces cell death.	Gramont et al. (2000), Parker & Cheng (1990), Goldberg et al. (2004), Raymond et al. (1998)
FOLFIRI	Chemotherapeutic regimen involving the use of folinic acid, 5-FU and irinotecan to interfere with DNA uncoiling during DNA replication which ultimately induces cell death.	Klein et al. (2002), Douillard et al. (2000), Saltz et al. (2000)
Growth factor receptor inhibitors (*e.g.*, Bevacizumab, Cetuximab)	Therapeutic agents designed to target specific pathways supporting cancer proliferation and formation of new blood vessels that allow the spread of mCRC.	Sherwood, Parris & Folkman (1971), Venook (2005)
Tyrosine kinase inhibitors (*e.g.*, Gefitinib, Erlotinib, Sorafenib)	Therapeutic agents designed to target tyrosine kinases that mediate downstream signalling events of mCRC.	Huang et al. (2004), Townsley et al. (2006), Wilhelm et al. (2006)

### Drug resistance in CRC

Despite advances in the diagnostics and treatments, the global age-standardised mortality rates of CRC remain high (8.9 per 100,000 population in both sexes) (Siegel et al., 2017; Rawla, Sunkara & Barsouk, 2019). This is mainly due to the development of resistance to the standard chemotherapeutic regimens (5-FU and L-OHP) or combinational treatments (FOLFOX and XELOX) (Swanton, 2012; Weeks et al., 2012; Pai et al., 2017). In such cases, targeted therapeutic agents such as growth factor receptor inhibitors and protein kinase inhibitors are combined with the standard treatments to improve drug efficacy and patients’ response rates (Cunningham et al., 2004; Heinemann et al., 2014). Nevertheless, CRC patients have also been reported to develop resistance against these targeted therapeutic agents (Chen et al., 2018a; Negri et al., 2019; Vitiello et al., 2019; Rimassa et al., 2019). Hence, cancer drug resistance represents a significant obstacle to the successful treatment of CRC patients.

Drug resistance occurs when a tumour has become insensitive to the prescribed drugs, leading to the emergence of drug-tolerant cancer persister cells which support the growth of cancer cells under treatment pressure (Ramirez et al., 2016; Russo et al., 2019). While intrinsic or primary drug resistance occurs before drug treatment, acquired or secondary drug resistance manifests itself as a gradual reduction in drug efficacy against CRC (Lippert, Ruoff & Volm, 2008). Among the factors culminating in drug resistance, overexpression of ATP-binding cassette (ABC) transporters has been identified as the main driver. ABC transporters function to mediate the efflux of drugs from the tumours, leading to reduced drug concentration and drug efficacy (Giacomini et al., 2010; Hu et al., 2016). On top of that, the development of drug resistance has been attributed to genetic and epigenetic alterations, such as the (i) overexpression and gain-of-function of oncogenes (*e.g.*, epidermal growth factor receptor (EGFR), Kirsten rat sarcoma virus (KRAS)) (Lièvre et al., 2006; Misale et al., 2014; Wang et al., 2019; Wang, Zhang & Chen, 2019), (ii) loss-of-function of tumour suppressor genes (*e.g.*, p53, phosphatase and tensin homolog (PTEN)) (Boyer et al., 2004; Frattini et al., 2007; Sartore-Bianchi et al., 2009), (iii) under-expression of cell signalling regulator (*e.g.*, thymidine phosphorylase) (Meropol et al., 2006), and (iv) the change in the binding site of drug target (*e.g.*, topoisomerase I) (Gongora et al., 2011). In addition, the evolution of CRC subclones further complicates CRC treatment due to the limited ability of the cancer therapeutics to counteract the diverse drug resistance mechanisms present in the heterogeneous cancer subpopulations (Molinari et al., 2018; Wang et al., 2019; Wang, Zhang & Chen, 2019).

It has increasingly been acknowledged that various molecular mechanisms contribute to cancer drug resistance, among which the dysregulation of signalling pathways has been shown to play critical roles (Nisar et al., 2020). As such, the study of cell signalling pathways can provide valuable insights into the cancer biology of drug-resistant CRC and improve the treatment strategies (Wan et al., 2020). Previous studies have attributed four major signalling pathways (MAPK, PI3K/PKB, Wnt/β-catenin and Notch) to the development of resistance against CRC treatment (Li et al., 2011; Corcoran et al., 2012; Xu et al., 2017; He et al., 2018). There are significant efforts focusing on delineating tumour evolution and the underlying molecular mechanisms of drug resistance linked to these signalling pathways. Numerous studies have revealed that genetic mutations and/or epigenetic alterations of these pathways contribute to drug resistance (Normanno et al., 2015; Jeantet et al., 2016; Yamada et al., 2020). Apart from that, recent evidence also indicates that resistance of the tumour cells involves highly complex and tightly controlled crosstalk between different signal transduction pathways (Duong et al., 2018). Additionally, emerging findings suggest that signalling related to tumour microenvironment (TME), metabolic reprogramming and gut microbiome are also associated with the development of drug resistance (Lotti et al., 2013; Endo et al., 2020). A summary of the molecular alterations and clinical implications associated with the treatment of CRC is provided in Table 2.

**Table 2:** Molecular alterations and clinical implications associated with the dysregulation of targeted signalling pathways in multidrug-resistant CRC.

Therapeutic agent	Targeted signalling pathway	CRC mutational status	Molecular alteration	Clinical implication	Reference
Anti-EGFR antibodies (cetuximab and panitumumab) alone or in combination with chemotherapy	MAPK pathway	Wild-type KRAS	*KRAS*, *NRAS*, *BRAF* and *PI3KCA* mutations	•Poor prognosis for overall survival •Low response rate to anti-EGFR therapy	De Roock et al. (2010), Diaz et al. (2012)
Anti-EGFR antibodies (cetuximab, panitumumab, SYM004, MM151, trastuzumab, pertuzumab and duligotuzumab) alone or in combination with chemotherapy	MAPK pathway	Wild-type KRAS, NRAS, BRAF and PI3KCA	•*HER2* gene amplification and activating mutations •Sustained signalling of PI3K/PKB and MAPK pathways	•Poor therapeutic response •Oncogenic transformation of colon epithelial cells	Kavuri et al. (2015), Belli et al. (2019)
RAF inhibitor (vemurafenib)	MAPK pathway	BRAF(V600E)	Feedback activation of EGFR	Treatment failure	Prahallad et al. (2012)
Combined RAF inhibitors (vemurafenib and cetuximab or vemurafenib and selumetinib)	MAPK pathway	BRAF(V600E)	Reactivation of MAPK pathway	Tumour relapses	Ahronian et al. (2015)
RAF inhibitors (GDC-0879 and vemurafenib)	MAPK pathway	BRAF(V600E)	RAF dimerization and MEK/ERK phosphorylation	Enhanced tumour growth	Hatzivassiliou et al. (2010)
Vemurafenib	MAPK pathway	KRAS(G13D)	Activation of ERK leads to the activation of Hippo and Rho pathways	Cancer metastasis	Kubiniok et al. (2017)
Chemotherapeutic drug (oxaliplatin)	MAPK pathway	Not applicable	miRNA-625-3p-mediated downregulation of MAP2K6	Cancer progression due to reduced apoptosis.	Rasmussen et al. (2016)
Combinational chemotherapeutic drugs (FOLFOX and FOLFIRI)	PI3K/PKB pathway	Not applicable	*PIK3CA* mutations (E545K, E542K and E545D on exon 9; H1047R and H1047L on exon 20)	LGR5+ CRC stem cells survival and proliferation	Wang et al. (2018)
Cetuximab	PI3K/PKB pathway	Wild type KRAS and BRAF	*PIK3CA* mutations on exon 19 (K944N, V955I, F930S, V955G and K966E)	Decrease in progression-free survival	Xu et al. (2017)
Cetuximab and panitumumab	•MAPK pathway •PI3K/PKB pathway	Wild type KRAS	*BRAF, NRAS, PTEN* and *PIK3CA* mutations	•Poor prognosis for overall survival •Cancer metastasis	Sartore-Bianchi et al. (2009), Laurent-Puig et al. (2009), De Roock et al. (2010)
NVP-BEZ235 (dual PI3K/MTOR inhibitor)	•MAPK pathway •PI3K/PKB pathway	Not applicable	*KRAS and PIK3CA* mutations leads to additive activation of PI3K/PKB pathway	Suppression of BIM-induced apoptosis.which leads to cancer survival	Kim et al. (2013)
Chemotherapeutic drug (paclitaxel)	PI3K/PKB pathway	Not applicable	miR-29a-mediated PTEN inhibition	Reduction in drug sensitivity suppress apoptosis which supports cancer growth	Yuan et al. (2018)
Chemotherapeutic drug (doxorubicin)	PI3K/PKB pathway	Not applicable	miR-29a-mediated P-gp inhibition and upregulation of PTEN	Enhanced drug sensitivity which thwart cancer growth	Shi et al. (2020)
Chemotherapeutic drug (5-FU)	PI3K/PKB pathway	Not applicable	miR-543-mediated PTEN inhibition	Reduced drug sensitivity which supports cancer growth	Liu, Zhou & Dong (2019)
Chemotherapeutic drug (vincristine)	Wnt/β-catenin pathway	Not applicable	Overexpression of Dvl1-3 leads to β-catenin/TCF-induced transcription of ABC transporters (P-gp, MRP2 and BCRP)‘and anti-apoptotic proteins (Survivin and Bcl-2)	CRC is protected from Vincristine-induced apoptosis which drives cancer growth	Zhang et al. (2017b)
5-FU and oxaliplatin	Wnt/β-catenin pathway	Not applicable	Overexpression of LINC00152 inhibits CK1α-dependant β-catenin phosphorylation	•Cancer metastasis •Expression of EMT markers	Yue et al. (2016), Yue et al. (2018), Bian et al. (2017)
5-FU	Wnt/β-catenin pathway	Not applicable	miR-30-5p-mediated inhibition of USP22 and Wnt target genes (*Axin2* and *c-Myc*)	Suppression of cancer stemness and chemoresistance	Ning et al. (2014), Jiang et al. (2019)
5-FU and oxaliplatin	Wnt/β-catenin pathway	Not applicable	LncRNA CRNDE-mediated repression of miR-181a-5p promotes β-catenin/TCF transcriptional activity	CRC cell proliferation and chemoresistance	Han et al. (2017)
Small-molecule multi kinase inhibitor (regorafenib)	Notch pathway	Not applicable	Upregulation of Notch-1 and the target genes (*HES1* and *HEY1*)	CRC cell proliferation due to reduced sensitivity to Regorafenib	Mirone et al. (2016)
Anti-VEGF antibody (bevacizumab)	Notch pathway	Not applicable	Upregulation of NICD	Cancer stemness	Negri & Ardizzoni (2015), Negri et al. (2019)
5-FU	Notch pathway	Not applicable	HES1-mediated overexpression of ABC transporters (ABCC1, ABCC2 and P-gp1) with depressed E-cadherins and elevated N-cadherins	Tumour relapses	Gao et al. (2014), Sun et al. (2017)
Chemotherapeutic drug (methotrexate)	•Notch pathway •Wnt/β-catenin pathway	Not applicable	Dvl-3-related Wnt and Notch crosstalk.	Cancer stemness	Zhao et al. (2020)
5-FU and Irinotecan	•Notch pathway •KRAS/Erk/ADAM pathway	KRAS(G12D, G12A, G13D, Q61L)	Aberrant Jagged1 processing leads to sustained Jag1-ICDs-mediated intrinsic reverse signalling.	Cancer progression and chemoresistance	Van Schaeybroeck et al. (2011), Pelullo et al. (2019)
5-FU	Notch pathway	Not applicable	miR-139-5p-mediated inhibition of Notch-1 and downstream multidrug-resistant genes (MRP-1 and BCL-2)	Increased sensitivity to 5-FU	Liu et al. (2016)
5-FU	Notch pathway	Not applicable	miR-195-5p-mediated inhibition of Notch-2 and RBPJ	Inhibition of cancer stemness and 5-FU resistance	Jin et al. (2018)
5-FU	Notch pathway	Not applicable	miR-34a-mediated ABCG2 inhibition	Enhanced chemosensitivity to 5-FU	Xie, Chen & Fang, (2020a), Xie et al. (2020b)

 In this review article, we attempt to summarize the gap in knowledge in understanding the link between modulation of the signalling mechanisms due to diverse exogenous and endogenous factors with drug resistance in CRC. We aim to provide current updates related to the dysregulation of the four selected signal transduction pathways and their roles in conferring drug resistance in CRC. In addition, future perspectives pertinent to the involvement of other signalling pathways and resistance mechanisms due to TME, metabolic reprogramming and gut microbiome are also discussed.

### Survey methodology

To ensure a thorough and unbiased coverage of the literature, we searched fthe PubMed database for published articles written in English from 1990 until present. The search strings include “colorectal cancer AND (crosstalk OR communication) AND (signalling OR pathway) AND therapy resistance”, “colorectal cancer AND (monotherapy OR combinational therapy) AND drug resistance AND MAPK pathway”, “colorectal cancer AND (monotherapy OR combinational therapy) AND drug resistance AND PI3K pathway”, “colorectal cancer AND (monotherapy OR combinational therapy) AND drug resistance AND Wnt pathway”, “colorectal cancer AND drug resistance AND Notch”, and “colorectal cancer AND (MAPK OR PI3K OR Wnt OR Notch) AND drug resistance”. The searches were performed by two authors independently of each other. The abstracts of these articles were then assessed to exclude papers that were not relevant to the scope of this review. This review paper is intended for scientist who work in the same scientific area as well as other readers in the field of molecular and cancer biology in general.

### Regulation of signalling pathways associated with drug resistance in CRC

#### Mitogen-activated protein kinase (MAPK) pathway

**Figure 1:**
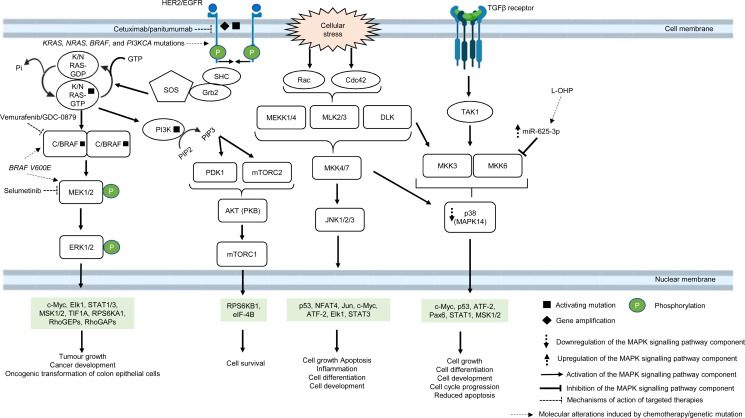
Deregulation of the canonical MAPK signalling pathway during CRC treatment. Region highlighted green represents substrates for MAPKs that regulate biological processes in CRC cells treated with drug therapy.

The MAPK pathway is mediated by mitogen-activated protein kinases (MAPKs), which comprise a family of serine/threonine-specific protein kinases that regulate a variety of cellular processes and play crucial roles in the pathogenesis of many diseases such as cancer, infection, inflammatory and autoimmune diseases (Kim & Choi, 2010). The MAPK family is divided into several subgroups. Conventional MAPKs are (i) extracellular signal-regulated kinase 1 and 2 (ERK1/2), (ii) c-Jun N-terminal kinases (JNKs), (iii) p38 (also known as MAPK14) and (iv) ERK5, all are summarised in Fig. 1 (Morrison, 2012). Atypical MAPKs comprise ERK3/4, ERK7/8 and Nemo-like kinase (NLK) (Cargnello & Roux, 2011). In the canonical ERK/MAPK signalling pathway, extracellular signals (*e.g.*, growth factors, stress, mitogens) bind to the receptors, most of which are receptor tyrosine kinases (RTKs) at the surface of the cell membrane, leading to auto-phosphorylation of growth factors receptors and recruitment of adaptor proteins (*e.g.*, growth factor receptor-bound protein 2 (GRB2) and son of sevenless 1 (SOS1)) (Morrison, 2012). This in turn results in the switching of the inactivated form of Ras-family GTPase (Ras in GDP bound form, Ras-GDP) to the active form of Ras-family GTPase (Ras in GTP bound form, Ras-GTP). The external signal is then transmitted *via* Ras-GTP to other downstream phosphorylation targets within the cytoplasm where the signalling cascade converges at the activation of a series of MAPKs, starting from MAPK kinase kinase (MAPKKK, *e.g.*, Raf1) followed by MAPK kinase (MAPKK, *e.g.*, MEK1/2) and MAP kinase (MAPK, *e.g.*, ERK1/2). Finally, the MAP kinase translocates to the nucleus to phosphorylate transcription factors (*e.g.*, c-Jun, STAT1, c-Myc) that regulate transcription of genes for different cellular processes (Wei & Liu, 2002; Fang & Richardson, 2005; Morrison, 2012) (Fig. 1).

In multidrug-resistant CRC, MAPK pathway is often reprogrammed, usually by the overexpression of RTKs, Ras and Raf; or gain-of-function mutations of Ras and Raf, which sustain the activity of MAPK signalling pathway upon treatment with MAPK and RTK inhibitors, 5-FU and oxaliplatin (Wan et al., 2004; Kavuri et al., 2015; Martinelli et al., 2017; Ressa et al., 2018). EGFR has been a favourable target for the treatment of mCRC since the last decade, mainly because they are highly expressed in most human tumours, including CRC (Yarden & Pines, 2012). In particular, monoclonal antibody targeting EGFR, such as cetuximab and panitumumab are widely used to treat mCRC patients due to their initial benefit of improving patients’ survival (Jonker et al., 2007; Vermorken et al., 2008; Pirker et al., 2009). However, some studies have shown that anti-EGFR based therapy may not be effective in treating mCRC, indicating that a subset of CRC is resistant to the anti-EGFR treatment (Bokemeyer et al., 2009; Van Cutsem et al., 2009). Common molecular mechanisms associated with the resistance are KRAS, NRAS, BRAF and PI3KCA mutations (De Roock et al., 2010; Diaz et al., 2012). In CRC which is quadruple wild-type for KRAS, NRAS, BRAF and PI3KCA genes, HER2 gene amplification and activating mutations at the phosphorylation sites of the catalytic domain have been shown to bypass EGFR blockade by activating a compensatory signalling mechanism for cell survival (Kavuri et al., 2015; Belli et al., 2019). It has been reported that HER2 could form heterodimers with either EGFR or ERBB3 with consequent activation of ERK and Akt signalling respectively, in which the latter has been shown to promote anti-EGFR resistance (Zhang et al., 2014a; Zhang et al., 2014b). Besides, it has been found that aberrant ERBB2 activation could result in the stimulation of ERK 1/2 signalling that mediates cetuximab resistance (Yonesaka et al., 2011).

B-Raf is a MAPKKK that mediates cell growth and differentiation *via* the ERK/MAPK subfamily of MAPK pathway, in response to growth factors and mitogens (Morrison, 2012). B-Raf mutations, more often BRAF V600E, occur in approximately 8% of CRC and are associated with poor prognosis (Davies et al., 2002; Richman et al., 2009). BRAF V600E mutation leads to conformational changes at the catalytic domain which renders B-Raf constitutively active, independent of Ras-GTP activation and dimerization with Raf-1 (also known as C-Raf) (Durrant & Morrison, 2018). This results in prolonged phosphorylation and activation of MEK1/2 and ERK1/2 kinases which, in turn, activates downstream substrates that mediate cell growth and survival (Fang & Richardson, 2005). It has been reported that targeting BRAF V600E using mono-therapeutic agents, such as vemurafenib (a B-Raf inhibitor, also known as PLX4032) which binds to the ATP-binding site of BRAF V600E to inhibit its activity, shows limited therapeutic response in CRC (Prahallad et al., 2012). This is because targeting BRAF V600E results in feedback activation of EGFR characterised by enhanced phosphorylation of RAS and CRAF upstream of MAPK pathway and downstream activation of RAF, MEK and ERK (Corcoran et al., 2012). In order to circumvent resistance to B-Raf inhibition, B-Raf inhibitor is used in combination with EGFR inhibitor or MEK inhibitor or both which were initially shown to offer therapeutic benefit of at least 12% response rate to the drugs and improve the suppression of ERK/MAPK pathway (Bendell et al., 2014; Corcoran et al., 2014; Tabernero et al., 2014). Despite the initial success in suppressing B-Raf resistance using the multi-target approach, there is compelling evidence that BRAF V600E mutant CRC patients could also develop resistance to the new treatment (Oddo et al., 2016). Ahronian et al. (2015) has shown that the reactivation of the MAPK pathway confers cross-resistance to the combined RAF/EGFR or RAF/MEK inhibition in BRAF-mutant CRC and further demonstrated that the use of ERK inhibitor could overcome the resistance by suppressing the MAPK signalling.

On the other hand, it has been reported that treatment using ATP-competitive inhibitors produces opposing mechanisms of action that is dependent on the cellular context and genotype of the tumour. It was found that RAF inhibitors effectively block MAPK pathway in BRAF V600E cells but activate the MAPK pathway in wild-type BRAF tumours by inducing RAF dimerization and MEK/ERK phosphorylation leading to enhanced tumour growth, suggesting that other strategies to block RAF activation are needed to improve the treatment efficacy (Hatzivassiliou et al., 2010). Furthermore, RAS mutant tumours are also known to exhibit poor response to RAF inhibitors. A time-course phosphoproteomic analysis of vemurafenib-treated RAS mutant CRC cell lines has found potential cross-talk between ERK signalling with Hippo and Rho pathways and revealed novel functional targets downstream of ERK (Kubiniok et al., 2017).

Apart from post-translational regulation of proteins as a regulatory checkpoint for cellular signalling, microRNAs (miRNAs) also exhibit a functional role in the regulation of MAPK pathway in CRC drug resistance (Rasmussen et al., 2016; Angius et al., 2019). A previous miRNA profiling study has uncovered the link between high expression of miRNA-625-3p and poor clinical response towards oxaliplatin-based therapy (Arango et al., 2004; Rasmussen et al., 2013). Mechanistically, it has been demonstrated that miRNA-625-3p mediates oxaliplatin resistance by targeting MAPK kinase MAP2K6 and abrogates MAPK14 signalling, leading to increased cell cycle progression and reduced apoptosis (Rasmussen et al., 2016).

As one of the most frequently altered signalling pathways and its important roles in CRC drug resistance, MAPK pathway represents a promising target for cancer therapy. Significant progress has been made on the development of therapeutics targeting MAPK kinases with considerable clinical success (Bendell et al., 2014). Notably, the combination of BRAF and MEK inhibitors has been shown to improve response rates and may offer potential therapeutic benefit in BRAF-mutated CRC (Corcoran et al., 2014). Nevertheless, it is clear that we are still far from any complete understanding of the MAPK pathway. Moreover, the emergence of new resistance mechanisms prompts more further research to provide a deeper understanding on the complex regulation and interconnectivity of the underlying biological processes to overcome resistance and increase therapeutic efficacy.

#### Phosphoinositide 3-kinase (PI3K)/protein kinase B (PKB, also known as AKT) pathway

**Figure 2:**
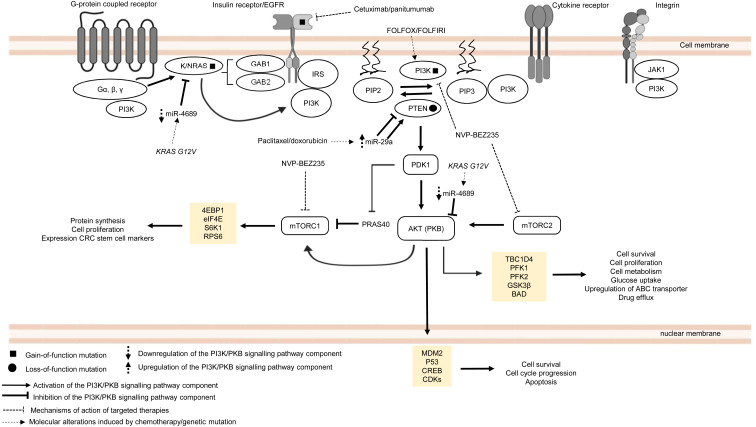
Deregulation of the canonical PI3K signalling pathway during CRC treatment. Region highlighted yellow represents substrates for PKB/AKT that regulate biological processes in CRC cells treated with drug therapy.

The PI3K/PKB pathway regulates cell metabolism, cell growth and cell survival. In normal condition, PI3K/PKB pathway is activated by four major sensors upstream of the pathway, namely (i) receptor tyrosine kinases (RTKs) which bind to growth factors (Hemmings, 1997), (ii) cytokine receptors (Chang et al., 2003), (iii) G protein-coupled receptors (GPCRs) that are activated by various biological molecules (Murga et al., 1998), and (iv) integrins which detect cell–cell or cell–matrix communication (Su et al., 2007). Upon ligand binding, these receptors, together with their cofactors, will activate PI3K family proteins. There are three classes of PI3K family proteins, among which only class I PI3Ks and the signalling networks they regulate are covered in this review (Fig. 2). Information about Class II and Class III PI3Ks and their roles in cellular signalling are covered in other reviews or journal articles (Falasca & Maffucci, 2012; Okkenhaug, 2013; Backer, 2016; Hawkins & Stephens, 2016). Within the class I PI3K subfamily itself, there are four catalytic isoforms (p110α, p110β, p110γ and p110δ encoded by *PIK3CA*, *PIK3CB*, *PIK3CG*, and *PIK3CD* respectively) which catalyse the phosphorylation of phosphatidylinositol-4,5-bisphosphate (PI(4,5)P_2_) to phosphatidylinositol (3,4,5)-trisphosphate (PI(3,4,5)P_3_) or PIP_3_. p110α and p110β are expressed ubiquitously while p110γ and p110δ are expressed in immune cells. Each catalytic isoform forms a dimer with a regulatory subunit that controls the activity and subcellular localisation of the PI3K complex (Fig. 2). In response to specific external stimuli, PIP_3_, which acts as a secondary messenger, will recruit cytoplasmic proteins with PIP_3_ binding domains (typical examples of which are phosphoinositide-dependent kinase-1 (PDK1) and PKB) to specific cell membrane locations. Shortly after the transmission of the signal to downstream effectors, PIP_3_ is then metabolised by phosphatase and tensin homolog (PTEN), which is a tumour suppressor that negatively regulates the PI3K signal by removing 3′-phosphate from PIP_3_ (Danielsen et al., 2015; Fruman et al., 2017). Activation of PKB is a two-step process, whereby PDK1 phosphorylates PKB on threonine-308 to partially activate PKB (Alessi et al., 1997), followed by phosphorylation of PKB by mTORC2 on serine-473 to fully activate PKB (Sarbassov et al., 2005). The activated PKB will subsequently regulate the phosphorylation of the target substrates (the most well-known examples are glycogen synthase kinase 3 beta (GSK3β), BCL2-antagonist of death (BAD), mouse double minute homolog 2 (MDM2), forkhead box O (FOXO) and mechanistic target of rapamycin complex (mTORC1)) which mediate important cellular functions, such as glucose uptake, protein synthesis, cell survival and cell cycle progression (Manning & Cantley, 2007). The activity of PKB is also negatively regulated by protein phosphatase 2A (PP2A), PH domain leucine-rich repeat protein phosphatase 1/2 (PHLPP1/2) and carboxyl-terminal modulator protein (CTMP) (Hemmings & Restuccia, 2012) (Fig. 2). In multidrug-resistant CRC, PI3K signalling is prolonged by *PIK3CA* mutations, null mutation of PTEN, and RAS mutations upon treatment with standard chemotherapeutic drugs and targeted therapeutic agents (Laurent-Puig et al., 2009; De Roock et al., 2010; Kim et al., 2013; Hamada, Nowak & Ogino, 2017).

*PIK3CA* is regarded as one of the most frequently mutated genes in CRC, which accounts for approximately 10–30% of all CRC cases (Samuels et al., 2004; Velho et al., 2005; Hamada, Nowak & Ogino, 2017). It is reported that non-random somatic mutations occurring in the coding region, mainly exon 9 (helical domain) and exon 20 (catalytic domain), have heightened basal PI3K and PKB activities which then promote cancer progression (Kang, Bader & Vogt, 2005; Ikenoue et al., 2005). Furthermore, it has been shown that *PIK3CA* mutations (E545K, E542K and E545D on exon 9; H1047R and H1047L on exon 20) could mediate resistance to standard chemotherapy (FOLFOX and FOLFORI) by inducing phosphorylation of PKB and expression CRC stem cell markers (LGR5) *via* sustained PI3K signalling, thereby promoting cancer cell survival and proliferation (Wang et al., 2018). Interestingly, *PIK3CA* mutations have also been found to mediate acquired cetuximab resistance in mCRC complementary to the previously reported RAS mutations. A recent circulating tumour DNA sequencing analysis from mCRC patients has revealed five novel mutations on exon 19 of PIK3CA (K944N, V955I, F930S, V955G and K966E) that may potentially drive resistance to cetuximab *via* EGFR-mediated activation of the PI3K/PKB signalling pathway, suggesting that combined regimens of PI3K/mTOR inhibitors (PP242 and NVP-BEZ235) with anti-EGFR therapy may be beneficial to overcome the resistance (Xu et al., 2017).

Apart from *PIK3CA* mutations, there is increasing evidence of the emergence of mutations involving components of MAPK and PI3K/PKB signalling pathways when treated with targeted therapeutic agents (De Roock et al., 2010; Vitiello et al., 2019). Although anti-EGFR based therapy is commonly prescribed for KRAS wild-type CRC patients, clinical evidence has indicated that the KRAS mutation status alone is insufficient to predict therapeutic response to the therapy (Allegra et al., 2009). Retrospective cohort studies of CRC cases have identified several key players in the EGFR signalling pathway (which is a signalling network shared by both MAPK and PI3K/PKB pathways) that hinder the effectiveness of anti-EGFR monoclonal antibodies in KRAS wild-type CRC patients (Sartore-Bianchi et al., 2009; Laurent-Puig et al., 2009; De Roock et al., 2010). It has been previously reported that BRAF, NRAS, PTEN and PIK3CA mutations are associated with the efficacy and clinical outcome of EGFR-targeted therapy but their exact roles in driving the resistance are still unclear (Laurent-Puig et al., 2009; De Roock et al., 2010). Surprisingly, KRAS mutations also negatively affect the outcome of treatment against *PIK3CA* mutant CRC. Kim et al. (2013) have shown that *KRAS* and *PIK3CA* mutations attenuate sensitivity to treatment with a dual inhibitor of PI3K and mTOR by suppressing BIM-induced apoptosis *via* activation of PI3K/MTOR pathway, leading to cell survival.

More recently, miRNAs have also been reported to control CRC pathogenesis *via* the modulation of PI3K/PKB pathway (Soleimani et al., 2019). Based on previous studies, it has been shown that miR-29a could induce or suppress tumour progression in drug-resistant cancer cells (Zhong et al., 2013; Liu et al., 2018). Yuan et al. (2018) showed that higher level of miR-29a is expressed in CRC cell lines resistant to paclitaxel which resulted in downregulation of PTEN and upregulation of phosphorylated AKT. This suggests that miR-29a has a regulatory function to PI3K/PKB pathway *via* inhibition of PTEN which reduces drug sensitivity and supports cancer growth. In contrast, miR-29a could potentially reduce P-gp-mediated chemoresistance *via* modulation of PTEN and P-gp expression in doxorubicin-resistant CRC cell lines. This means that miR-29a exerts tumour suppressive function by inhibiting membrane transporter activity through PI3K/PKB pathway (Shi et al., 2020). These seemingly contradictory roles of miR-29a have also been observed in other malignancies. In the context of regulation of drug resistance, miR-29a has been reported to increase sensitisation to gemcitabine in pancreatic cancer cells (Kwon et al., 2016) as well as sensitization to tamoxifen in breast cancer cells (Muluhngwi et al., 2017). On the other hand, miR-29a was shown to play a role in mediating adriamycin resistance in breast cancer cells *via* inhibiting the PTEN/AKT/GSK3β pathway (Shen et al., 2016). It is unclear whether the opposing results are due to heterogeneity in the cancer cells or that miR-29a could exhibit multifaceted functions in a context dependent manner. Nevertheless, these paradoxical findings demand the need for further work to clarify and elucidate the function of miR-29a more comprehensively. Similar to miR-29a, miR-543 has recently been identified as the mediator of chemoresistance in CRC cells, also by suppressing the expression of PTEN which activates PI3K/PKB pathway (Liu, Zhou & Dong, 2019). Interestingly, miR-4689 which has been identified as a negative regulator of KRAS and AKT is downregulated in KRAS mutant CRC cells and confers resistance to molecular-targeted therapy, suggesting that miR-4689 could be a promising therapeutic agent to control multidrug resistance in CRC *via* modulation of PI3K/PKB pathway and MAPK pathway (Hiraki et al., 2015).

Taken together, current evidence clearly shows that mutations of the MAPK/PI3K/PKB signalling pathway components are frequently observed in cancer drug resistance. However, the signalling mechanisms associated with these mutations are still not well elucidated which necessitate further investigation. Importantly, distinct molecular events that regulate drug resistance such as in KRAS wild-type and mutant CRC suggests the importance of identifying relevant drug targets in CRC with different mutational status. Moreover, future research should also focus on dissecting the link of miRNAs with cancer drug resistance and their underlying molecular mechanisms.

#### Wnt/*β*-catenin pathway

**Figure 3:**
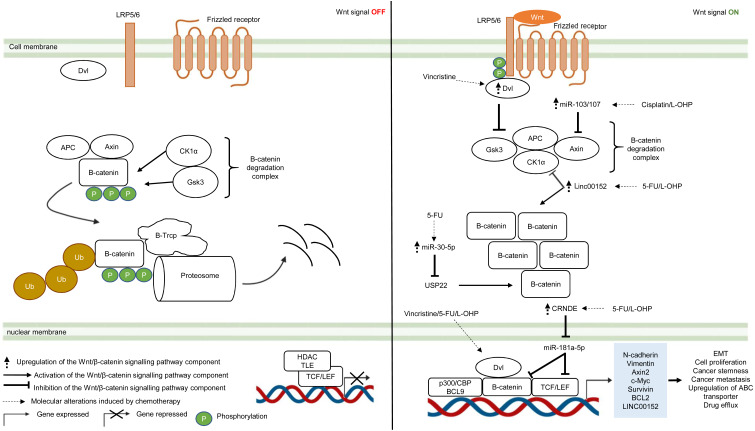
Deregulation of the canonical Wnt/β-catenin signalling pathway during CRC treatment. Left-hand-side of the figure describes signalling events in the absence of the Wnt signal (OFF). Right-hand-side of the figure describes signalling events in the presence of the Wnt signal (ON). Region highlighted blue represents Wnt target genes that regulate the biological processes of CRC cells treated with drug therapy.

The Wnt/β-catenin pathway is an evolutionarily conserved system that regulates cell development, cell differentiation, cell proliferation and cell migration. The Wnt/β-catenin pathway can be grouped into β-catenin-dependent Wnt pathway (canonical Wnt pathway) and β-catenin-independent Wnt pathway (non-canonical Wnt pathway) which are further divided into the planar cell polarity Wnt pathway and the Wnt/Ca^2+^ pathway (Komiya & Habas, 2008). The canonical Wnt/β-catenin pathway is made up of the membrane proteins, degradation complex and β-catenin protein. In the absence of Wnt ligands, the degradation complex which comprises of adenomatous polyposis coli (APC), Axin, GSK3 and CK1α is formed through phosphorylation of Axin and APC by GSK3 and casein kinase 1α (CK1α). As a result, β-catenin is ubiquitinated by E3-ligase protein βTrCP (β-transducin repeats-containing proteins) through phosphorylation and targeted for proteasomal degradation (Van Kappel & Maurice, 2017) (Fig. 3). In the presence of Wnt ligands, lipoprotein receptor-related protein 5/6 (LRP5/6) co-receptor and Frizzled (Fzd) receptor are activated which leads to phosphorylation of LRP5/6 co-receptors and binding of adaptor protein disheveled (Dvl) to the phosphorylated LRP5/6. This is followed by the recruitment of the remaining degradation complex components to the Fzd-LRP5/6 complex to inactivate the degradation complex (Janda et al., 2012) (Fig. 3). The molecular mechanism for Wnt-mediated degradation complex inactivation is still heavily disputed due to conflicting findings on the inhibition of GSK3 in the presence of Wnt signal. Several models have been proposed: (1) blockade of GSK3 catalytic site by binding to the phosphorylation motif of LRP5/6 (Wu et al., 2009). (2) Wnt-mediated dissociation of APC from GSK3 (Valvezan et al., 2012). (3) sequestration of GSK3 in endosomal vesicles through endocytosis of Fzd-LRP5/6 complex (Taelman et al., 2010). Disruption of the degradation complex integrity in the presence of Wnt signal promotes stabilisation of β-catenin, leading to accumulation of newly synthesised β-catenin in the cytoplasm and their subsequent translocation to the nucleus. Interestingly, it is also known that, in the presence of Wnt signal, the degradation complex could remain intact to target β-catenin for degradation *via* phosphorylation, but ubiquitination of β-catenin is impaired which inhibits its degradation by the degradation complex (Gerlach et al., 2014). Within the nucleus, β-catenin binds to T-cell factor/lymphoid enhancing factor (TCF/LEF) which are the transcription factors that activate Wnt-responsive genes required for cell growth and survival (*e.g.*, c-Myc, Cyclin D1). In addition, β-catenin interacts with TCF/LEF to recruit transcriptional co-activators (p300/CBP and BCL9) to the transcription factors to activate gene expression (Kretzschmar & Clevers, 2017; Taciak et al., 2018) (Fig. 3). In multidrug-resistant CRC, Wnt/β-catenin pathway is reprogrammed by overexpression of Dvl protein and non-coding RNAs that interfere with the activities of downstream signalling mediators (Yue et al., 2016; Han et al., 2017; Bian et al., 2017; Zhang et al., 2017b; Jiang et al., 2019).

Dysregulation in the key Wnt/β-catenin pathway components such as upstream regulator (Dvl protein), β-catenin degradation complex and its downstream targets (β-catenin and TCF/LEF) instigate tumour progression in many types of cancer (Van Kappel & Maurice, 2017). Moreover, recent studies indicate that aberrant Wnt/β-catenin signalling could trigger anti-cancer drug resistance (Zhang et al., 2017a). Zhang et al. (2017b) reported that DVL1-3 proteins are overexpressed in CRC resistant to vincristine (a chemotherapeutic drug that interferes with microtubule synthesis leading to cell cycle arrest) which results in overexpression of ABC transporters (P-glycoprotein (P-gp), MRP2 and BCRP) and anti-apoptotic proteins (Survivin and Bcl-2). Contrary to previous findings which suggest that DVL promotes β-catenin accumulation and subsequent translocation to the nucleus, it was found that DVL1-3 translocate to the nucleus and bind to β-catenin to form a transcriptional complex, independent of *β*-catenin accumulation and nuclear translocation (Gao & Chen, 2010; Shang, Hua & Hu, 2017). Moreover, the study also showed that silencing DVL1-3 could re-sensitise CRC cells to vincristine, 5-FU and oxaliplatin, suggesting that DVL could be a potential therapeutic target in multidrug-resistant CRC.

It has been increasingly recognised that long non-coding RNA (lncRNA, a non-coding regulatory RNA with greater than 200 nucleotides in length) regulates Wnt/β-catenin pathway in multidrug-resistant CRC (Ma et al., 2016; Lu et al., 2017). Recent studies have shown that lncRNA cytoskeleton regulator RNA (CYTOR, also known as LINC00152) is overexpressed in CRC which confers resistance to oxaliplatin-induced apoptosis (Yue et al., 2016). In addition, elevated expression of LINC00152 is also observed in CRC that gives rise to 5-FU resistance and cancer metastasis (Bian et al., 2017). However, the regulatory mechanism of LINC00152 in the Wnt/β-catenin pathway of mCRC is still unknown (Yue et al., 2016). In a recent study, Yue et al. (2018) demonstrated that LINC00152 competitively binds to β-catenin to prevent CK1α from phosphorylating β-catenin. As a result, β-catenin accumulates and translocates to the nucleus to activate the expression of epithelial-mesenchymal transition (EMT) markers (N-cadherin and Vimentin) which are hallmarks of metastatic cancer. Reciprocally, β-catenin/TCF4 transcriptional complex promotes the expression of LINC00152 to sustain Wnt/β-catenin signalling in mCRC.

Likewise, miRNA is also known to influence cancer growth and the sensitivity of cancer cells towards anti-cancer drugs (Jansson & Lund, 2012; Piletič & Kunej, 2016). Jiang et al. (2019) have demonstrated that overexpression of miR-30-5p negatively regulates the expression of Wnt/β-catenin pathway target genes (Axin2 and c-Myc) and inhibits chemoresistance in CRC cells by targeting ubiquitin-specific peptidase 22 (USP22). Mechanistically, it has been reported that USP22 induces β-catenin nuclear localisation and upregulates FoxM1 expression to promote G1/S cell cycle transition and cell proliferation (Ning et al., 2014). In another study conducted by Chen et al. (2019), miR-103/107 has been shown to repress the activity of Axin2 leading to sustained activation of Wnt/β-catenin signalling that potentiates cancer stemness and chemotherapeutic resistance. Nevertheless, despite that non-coding RNAs such as lncRNA and miRNA are known to promote resistance to therapeutic agents in CRC, the interaction network between LncRNA and miRNA is not well defined (Gao et al., 2019). Han et al. (2017) has reported that lncRNA Colorectal Neoplasia Differentially Expressed (CRNDE) binds to miR-181a-5p to repress its expression, resulting in increased levels of its downstream targets β-catenin and transcription factor TCF4 in the Wnt/β-catenin signaling pathway. It was also demonstrated that CRNDE knockdown and miR-181a-5p overexpression inhibit Wnt/β-catenin signaling and could reduce chemoresistance and attenuate cell proliferation in CRC cells, suggesting that it could be a novel cancer therapeutic strategy.

Over the years, substantial efforts have been directed to studying the molecular mechanisms and functional effects of Wnt signalling pathway. Accumulating studies confirm the critical role Wnt signalling in drug resistance and convey important insights into its underlying mechanisms that confer resistance to different therapies. Notably, such knowledge could be potentially harnessed to facilitate the development of specific inhibitors or drug combinations to improve anticancer efficacy. Nevertheless, owing to the complexity of Wnt signalling, there are still numerous details remain be uncovered with regard to its connections to therapy resistance that warrant further investigation.

#### Notch pathway

**Figure 4:**
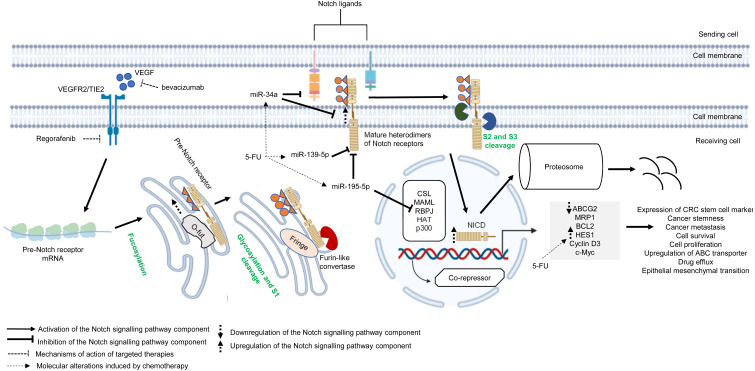
Deregulation of the canonical Notch signalling pathway during CRC treatment. Region highlighted grey represents Notch target genes that regulate the biological processes of CRC cells treated with drug therapy.

Similar to other signalling pathways (Wnt/β-catenin, Hedgehog (Hh), and transforming growth factor-beta (TGF-β)/bone morphogenic protein (BMP)), the Notch pathway is highly conserved across species and is known to control cell development, apoptosis, cell differentiation and proliferation (Artavanis-Tsakonas, Rand & Lake, 1999). Notch receptors (Notch 1–4) are synthesised as precursors from mRNAs (known as pre-Notch receptor) which then undergo fucosylation (a type of glycosylation) in the endoplasmic reticulum. In the Golgi apparatus, Notch receptors are further modified by enzymes (one typical example is Fringe) and cleaved at site 1 (S1) by furin-like convertase to induce heterodimerisation of Notch receptors (Siebel & Lendahl, 2017) (Fig. 4). At the cell surface, Notch receptors bind to Notch ligands (*e.g.*, Jagged1, Jagged2, Delta-like ligand 1 (Dll1), Delta-like ligand 3 (Dll3) and Delta-like ligand 4 (Dll4)) of neighbouring cells. This initiates subsequent cleavages of Notch receptors by ADAM10/17 metalloproteases and presenilin–γ-secretase enzyme complex at the outer side (site 2 (S2)) and inner side (site 3 (S3)) of the cell membrane (Bray, 2006) (Fig. 4). The end product of the proteolytic cleavages of Notch receptors known as Notch intracellular cleaved domain (NICD, an active form of the molecules which acts as transcriptional activators), travels to the nucleus to displace co-repressors (*e.g.*, recombining binding protein J-kappa (RBPJκ) or CSL) and interacts with transcriptional co-activators (*e.g.*, mastermind-like (MAML), histone acetyltransferase (HAT), p300), in order to activate the transcription of Notch target genes (*e.g.*, Hes and Hey family proteins, cyclin D3, c-Myc). Notch signalling is terminated when the intracellular domain of Notch (ICN or NICD) is targeted for proteasomal degradation through a ubiquitin pathway (Vinson et al., 2016; Siebel & Lendahl, 2017) (Fig. 4). In multidrug-resistant CRC, cross-regulation of signalling pathways, post-transcriptional regulation and overexpression of genes in the Notch signalling pathway are among the common mechanisms underlying the development of resistance towards targeted or chemotherapeutic regimens (Rodilla et al., 2009; Majidinia et al., 2018; Negri et al., 2019).

There is growing evidence that upregulation of Notch receptors, Notch ligands and Notch target genes could lead to the maintenance of CRC stem cell populations and the acquisition of metastatic phenotype which are strongly related to poor survival of CRC patients and drug resistance (Mohamed et al., 2019; Weber et al., 2019; Shaik et al., 2020). It has been previously reported that Notch-1 signalling pathway induces EMT in non-small cell lung cancer resistant cells with acquired resistance to EGFR tyrosine kinase inhibitor, suggesting that Notch signalling may contribute to cancer drug resistance (Xie et al., 2013). In another study, Mirone et al. (2016) has reported that CRC cells that are resistant to regorafenib (a small-molecule multi kinase inhibitor) showed significant upregulation of Notch-1 and the target genes (*HES1* and *HEY1*). The study demonstrated that knockdown of Notch-1 could partially restore the sensitivity to regorafenib and inhibit cell growth, indicating that Notch-1 may play a role in tumour resistance. In mCRC patients treated with bevacizumab-based therapy, high NICD expression was found to be associated with poorer response whereas no correlation was observed between Dll-4 expression and clinical response (Negri & Ardizzoni, 2015). In a follow-up study, Negri et al. (2019) has shown that high expression levels of NICD and CD44 are linked to cancer stemness in patients with advanced CRC treated with bevacizumab. Notably, the study demonstrated that NICDs (the functional components of Notch signalling pathway) instead of Dll-4 induce resistance to anti-angiogenic therapy in CRC *via* activation of Notch-induced regulation of colon cancer stem cells. Nevertheless, the functional roles of NICDs and CD44s in the CRC microenvironment during anti-angiogenic treatment are still unclear (Negri et al., 2019). HES1, which is a downstream target of Notch pathway and one of the important markers of CRC stem cells, is known to contribute to tumour relapses in CRC patients after 5-FU based chemotherapy, but the role of HES1 in chemoresistant CRC has not yet been elucidated (Gao et al., 2014). A recent study by Sun et al. (2017) has shown that HES1 modulates gene expression related to drug metabolism and EMT, notable overexpression of ABC transporters (ABCC1, ABCC2 and P-gp1) with depressed E-cadherins and elevated N-cadherins in CRC cell lines treated with 5-FU, supporting the crucial role of HES1 in promoting chemoresistance.

miRNAs are known to regulate Notch signalling pathway which results in various tumour pathology, such as metastasis, tumour relapses, cancer stemness and low survival rate (Majidinia et al., 2018; Khan et al., 2019). Recently, accumulating evidence also suggests that cross-regulation between miRNAs and Notch signalling pathway plays a critical role in cancer drug resistance. miR-139-5p is a tumour suppressor that has been found to be frequently downregulated in CRC (Shen et al., 2014). It has been reported that miR-139-5p targets Notch-1 and regulates its signal transduction to exert tumour suppressive effect in CRC (Zhang et al., 2014b). Furthermore, miR-139-5p/Notch-1 signalling has also been correlated with drug resistance in CRC. Liu et al. (2016) has shown that miR-139-5p sensitises CRC cells to 5-FU by inhibiting Notch-1 and its downstream multidrug-resistant genes (MRP-1 and BCL-2). Similarly, miR-195-5p is known to suppress cancer growth by inhibiting cell cycle progression, cell proliferation and cell migration (Luo et al., 2014). A recent study by Jin et al. (2018) has revealed that miR-195-5p could inhibit CRC cell stemness and 5-FU resistance by targeting the Notch signalling proteins Notch-2 and RBPJ, suggesting that miR-195-5p could be a potential therapeutic target in chemoresistance. On the other hand, a recent study by Xie et al. (2020b) reported that miR-34a could negatively regulate multidrug resistance protein ABCG2 *via* DLL1-mediated Notch signalling pathway and demonstrated that overexpression of miR-34a could overcome 5-FU resistance in CRC cells.

Overall, these findings suggest the critical role of Notch signalling pathway in cancer drug resistance. Notably, recent evidence indicates that Notch contribute to the maintenance of CRC stem cells and resistance to therapeutic agents, hence targeting Notch pathway may hold a promising prospect for cancer therapy. Thus, further studies are needed to elucidate the underlying mechanisms and the crosstalk between Notch and other signalling pathways to facilitate the design of better therapeutic approach.

### Crosstalk of signalling pathways

**Figure 5:**
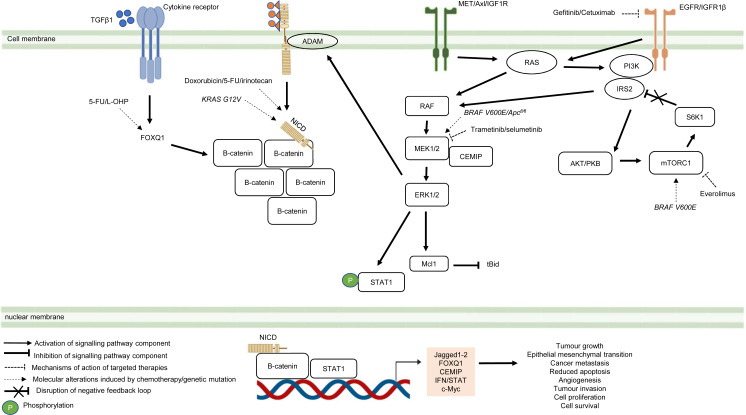
Crosstalk of signalling pathways during CRC treatment. Region highlighted orange represents substrates that regulate the biological processes of CRC cells treated with drug therapy.

Besides deregulated signalling events mediated by single pathway during CRC treatment, the pathological link between the crosstalk of signalling pathways and the acquisition of drug resistance in CRC has also been documented in numerous studies (Fig. 5) (Hiraki et al., 2015; Ahmed et al., 2015; Zou et al., 2017; Mesange et al., 2018). It has been shown that MAPK-mediated pathway interacts with other signalling pathways to induce drug resistance in CRC (Watanabe et al., 2011). Tyrosine kinase inhibitors such as gefitinib (EGFR inhibitor) desensitises CRC cells to the antitumour effect of the drugs by promoting the heterodimerisation of EGFR and IGF1Rβ, leading to cross-regulation of the IGFR1β and MAPK signalling pathways (Yang et al., 2011). The EGFR signalling pathway has also been reported to cooperate with the MAPK signalling pathways mediated by RTKs (MET, Axl, and IGF1R) to promote resistance against EGFR inhibitors such as cetuximab (Hu et al., 2016). In addition to MAPK-based signalling involving RTKs, KRAS-mediated activation of the MAPK signalling pathway in CRC has also been demonstrated to confer resistance to MEK inhibition by instigating STAT1 phosphorylation and the activation of IFN/STAT signalling (Sakahara et al., 2019). Additionally, research findings also suggest that BRAF V600E regulates the crosstalk of the KRAS-mediated MAPK signalling pathway with other signalling pathways in multidrug-resistant CRC (He et al., 2018; Duong et al., 2018). BRAF V600E promotes the expression of endosomal protein CEMIP *via* a β-catenin-dependant pathway that sustains ERK1/2 activation after MEK1 inhibition. The crosstalk between the Wnt/β-catenin and MAPK signalling pathways that involves CEMIP enhances the expression of c-Myc to promote cell survival (Duong et al., 2018). Inhibition of mTORC1 in BRAF V600E CRC has also been shown to disrupt the S6K1-IRS-2/PI3K negative feedback loop, leading to ERK-dependant Mcl-1 stabilisation which blocks apoptosis (He et al., 2018).

On the hand, several reports have shown that there is crosstalk between Notch and other signalling pathways that are involved in the development of chemoresistance. It has been demonstrated that Notch-1 signalling pathway could activate Wnt/β-catenin pathway by NICD-1-mediated translocation of β-catenin to the nucleus upon binding of Notch-1 to its ligands (Ishiguro et al., 2017). On the other hand, it has been reported that the activation of Notch signalling pathway in CRC cell lines is mediated by β-catenin through up-regulation of Jagged1 (Rodilla et al., 2009). Furthermore, it has been shown that β-catenin promotes the expression of Jagged2 in CRC and contributes to tumour resistance to chemotherapy through modulation of p21 (Vaish, Kim & Shim, 2017). Besides, it has been reported that Notch and Wnt pathways were both upregulated and associated with the development of chemoresistance in CRC cells by upregulating HES1 expression (Kukcinaviciute et al., 2018). Interestingly, a recent study has revealed that Dvl scaffold protein acts as a key regulator of the Wnt and Notch crosstalk (Zhao et al., 2020). Findings from the study highlighted that Dvl-3 might have a functional role in the acquisition of Methotrexate (MTX, an inhibitor of the dihydrofolate reductase (DHFR) enzyme) resistance and stem cell-like properties in CRC cell lines, although the mechanistic details of the resistance still remain unknown. Also, it has been found that Notch pathway could mediate chemoresistance *via* crosstalk with KRAS pathway. It has been previously reported that KRAS mutations regulate growth factor shedding following chemotherapy treatment *via* the MEK/Erk/ADAM17 signalling axis and contribute to drug resistance in CRC tumours (Van Schaeybroeck et al., 2011). In a recent study conducted by Pelullo et al. (2019), it has been demonstrated that Jagged-1-ICDs (Jag1-ICDs) are produced by aberrant Jagged1 processing *via* KRAS/Erk/ADAM pathway in CRC tumours with mutant KRAS. The study highlighted a novel role of Jag1-ICD beyond the canonical Notch signalling in mediating the oncogenic KRAS pathway, which promotes malignant behaviour and confers chemoresistance to CRC cells.

Besides the crosstalk between the Wnt/β-catenin and Notch signalling pathways, research evidence also suggests that TGF β1 induces the expression of FOXQ1 to promote the nuclear translocation of β-catenin. FOXQ1-mediated crosstalk of the Wnt/β-catenin and TGFβ1 signalling pathways results in resistance to chemotherapy drug-induced apoptosis, EMT and tumour invasion (Peng et al., 2015). In view of the importance of the crosstalk of signalling pathways in CRC drug resistance, future research therefore should aim to identify key regulators that mediate the such interaction to provide a holistic view of the resistance mechanism.

### Challenges and future perspectives

A variety of signalling pathways have also been demonstrated to induce drug resistance in CRC, other than the four signal transduction pathways discussed above (Lazzari et al., 2019; Kadioglu et al., 2021). For example, the activation of Hedgehog (Hh)-GLI pathway has been found to mediate the acquisition of chemoresistance *via* GLI-induced upregulation of ABC transporters in CRC (Po et al., 2020). It has also been reported that bone morphogenetic protein-2 (BMP-2) signalling activates STAT3 and promotes EMT and colon cancer stemness in CRC, which contribute to drug resistance (Kim et al., 2015). Likewise, studies have also suggested that Smad3/4 and IFN play important role in regulating multidrug-resistant CRC *via* STAT signalling (Moon et al., 2015; Sakahara et al., 2019). Some studies also feature other signalling events such as Hedgehog signalling in CRC during chemotherapy or molecular-targeted therapy, implying the underlying complexity of signalling mechanisms in multidrug-resistant CRC (Tang et al., 2018; Park et al., 2019).

On top of non-coding RNAs-based regulation of therapeutic resistance in CRC, epigenetic changes that involve DNA methylation and histone modifications have also increasingly been reported (Shen et al., 2018; Mahalakshmi, Husayn Ahmed & Mahadevan, 2018; Porcellini et al., 2018; Rezapour et al., 2019). For instances, hypermethylation of genes such as *MEIS2*, *SLFN11* and *B4GALT1* are associated with cancer progression and resistance to chemotherapy (cisplatin or oxaliplatin-based therapy) and anti-EGFR therapy (Baharudin et al., 2017; He et al., 2017; Picardo et al., 2019; Wang et al., 2019). Mechanistically, DNA methylation regulates the expression of miRNAs which influence the activity of signalling proteins in CRC with MSI-H to initiate anti-cancer drug resistance and cancer development (Shi et al., 2018). In addition, the regulatory function of histone methylation in multidrug-resistant CRC is also documented in studies that report the relationship between H3K27me3 level and oxaliplatin-induced apoptosis (Wang et al., 2020b), as well as the cancer-driving nature of histone methyltransferase SETDB1 in CRC resistant to cetuximab (Hou et al., 2020).

Besides cell-autonomous mechanisms of drug resistance, the association between tumour microenvironment (TME)-driven CRC pathogenesis and therapeutic failure has also been detailed in various studies (Hu et al., 2020; Ren et al., 2018; Hu et al., 2019; Jackstadt et al., 2019). Cancer-associated fibroblasts (CAFs), which constitute a main cellular component of the TME, have been identified as a key mediator of drug resistance in CRC by transferring exosomes to CRC cells (Kahlert & Kalluri, 2013; Herrera et al., 2018). Research evidence suggests that the transportation of exosomal miR-92a-3p from CAFs to CRC activates the Wnt/β-catenin pathway and inhibits mitochondrial apoptosis by targeting FBXW7 and MOAP1, contributing to cancer progression and chemotherapy resistance (Hu et al., 2019). The crosstalk between CAFs and CRC also involves the transfer of exosomal lncRNA H19 from CAFs to CRC cells (Ren et al., 2018). H19 activates the Wnt/β-catenin pathway by acting as a RNA sponge for miR-141, which inhibits the stemness of CRC cells (Ren et al., 2018). Correspondingly, exosomes derived from CRC cells also contain factors essential for reprogramming normal colonic fibroblast into CAFs which may in turn lead to chemoresistance in CRC (Rai et al., 2019). Potential strategies for CAFs-induced drug resistance in CRC include (i) suppressing the transformation of CAFs using small-molecule MSI-N1014 (Yadav et al., 2020), (ii) blocking tumoral IL1β-mediated signalling in normal colonic fibroblasts to thwart inflammatory CAF activation (Díaz-Maroto et al., 2021), and (iii) selective targeting of CAFs by engineered nanoparticles loaded with pro-apoptotic drug (Sitia et al., 2021). However, the failure to identify CRC subclones that mediate functional reprogramming of CAFs remains a big therapeutic hurdle for treating drug resistance in CRC that needs to be addressed. Furthermore, the components within the TME have also been reported to interact *via* multiple signal transduction pathways to confer drug resistance in CRC (Margolin et al., 2011). Paracrine signalling initiated by IL-17 derived from T_H_17 cells involves the crosstalk of the ERK pathway and NF-κB pathway to induce G-CSF expression, leading to the recruitment of immature myeloid cells to the TME and tumour resistance to anti-angiogenic therapy (Chung et al., 2013). Noncanonical TGFβ pathway interacts with PI3K/PKB pathway to sustain fibroblast activation during molecular-targeted treatment that inhibits IL-1β/TGF β signalling (Díaz-Maroto et al., 2019).

In recent decades, in addition to the conventional chemotherapy and targeted therapy, immunotherapy such as immune checkpoint blockade (ICB) has emerged as a promising strategy for cancer therapeutic. ICB works by inhibiting immune checkpoints to facilitate the activation of cytotoxic T cells and enhance anti-tumour immune response (Dosset et al., 2018; Woolston et al., 2019). The acquisition of resistance against ICB therapy, including those targeting cytotoxic T lymphocyte-associated protein 4 (CTLA-4), programmed death 1 (PD-1) and programmed death-ligand 1 (PD-L1), is well documented in numerous studies but the underlying mechanisms are still not well characterized (Gurjao et al., 2019; Liao et al., 2019). Molecular events associated with resistance against anti-PD-1 therapy include (i) suppressing IFN-γ-Stat1-Irf1 signalling in CRC and reducing cytotoxic tumour-infiltrating CD8^+^T cells by m^6^A methyltransferases-mediated downregulation of STAT1 (Wang et al., 2020a), (ii) the reduction of tumour suppressive myeloid cells *via* intracellular signalling initiated by myeloid receptor TREM2 (Molgora et al., 2020) and (iii) activating oncogenic myeloid-derived suppressor cells and regulatory T cells by enhanced transcription of the gene that encodes lactate transporter which induces lactate secretion (Li et al., 2020a; Li et al., 2020b). Emerging studies have also highlighted that several oncogenic signalling pathways that are involved in regulating immune response that renders resistance to ICB. Activation of the Wnt/β-catenin signalling pathway has been reported to be associated with a lack of T-cell infiltration within the tumour microenvironment in cancer patients (Spranger, Bao & Gajewski, 2015). This immunological defect was found to be mediated by decreased production of chemokine CCL4 that suppresses the recruitment of CD103+ dendritic cells, resulting in resistance to the immunotherapy. The interferon (IFN) signalling is another signalling pathway that has been implicated in resistance to ICB therapy. Patients that did not respond to anti-CTLA-4 antibody ipilimumab therapy was reported to harbour mutations in the interferon gamma (IFN-γ) pathway genes leading to the ability of the tumour cells to escape from T cells, which was identified as a primary resistance to anti-CTLA-4 therapy (Gao et al., 2016). Nevertheless, the roles of other signalling pathways that are involved in modulating the sensitivity and resistance to ICB are still largely unknown and require further studies. The current strategy to tackle anti-PD1 resistance involves reprogramming immunosuppressive myeloid cells to promote the expansion of tumour suppressive M1 macrophages (Lu et al., 2021).

In addition to CAFs and immune cells as the regulators of drug resistance in CRC, the emerging role of gut microbiota in the regulation of innate immune signalling and autophagy, which leads to chemoresistance in CRC, has also been reported. This suggests that crosstalk between different components of the TME has functional relevance in CRC development and clinical outcome, which prompts further investigation (Yu et al., 2017).

Tumour heterogeneity which is the cornerstone for the maintenance of cancer cell populations has been strongly correlated with therapy resistance (Schumacher et al., 2019). Mounting evidence has demonstrated the existence of various forms of tumour heterogeneity with the most frequently observed type being the genetic heterogeneity (Di et al., 2019; Loeb et al., 2019). Intra-tumour or inter-tumour genetic status was shown to influence the prognostic outcome and drug response and are determinants of resistance to anti-cancer therapy (Russo et al., 2016; De Angelis et al., 2016; Galofré et al., 2020; Bruun et al., 2020). Other types of tumour heterogeneity include cell type heterogeneity which is found between CRC subpopulations in the TME, as reported by Yoon et al. (2019) which showed that T-cell densities is highly variable in DNA mismatch repair-deficient tumour as compared to DNA mismatch repair-proficient tumour.

Tumour heterogeneity can be further classified into metabolic heterogeneity due to metabolic reprogramming in cells that contributes to disease development (Katoh, 2017). Metabolic reprogramming or alterations in the cellular metabolism is an important cancer hallmark to meet the increased energy and nutrient demand of malignant cells to promote tumour development. Notably, emerging evidence suggests that metabolic reprogramming could also contribute to resistance to antitumor drugs (Teng et al., 2017; Yu et al., 2021). The underlying mechanisms of metabolic adaptation during the development of drug resistance are still unclear, but available data implies that activation of oncogenic pathways are involved in the regulation of metabolic reprogramming implicated in resistance. Vellinga and colleagues (Vellinga et al., 2015) demonstrated that tumour metabolism was shifted from glycolysis towards oxidative phosphorylation in colon cancer cells that were exposed to chemotherapy to support tumour survival during treatment. It was discovered that the enhanced oxidative metabolism was mediated by histone deacetylase sirtuin-1 (SIRT1) and its substrate, the transcriptional coactivator PGC1α [239]. The study further showed that knockdown of SIRT1 or PGC1α sensitized the tumour cells to the drug treatment, suggesting that the SIRT1/PGC1α is a novel pathway of drug resistance that may be targeted for therapy. More recently, Barisciano et al. (2020) reported the role for miR-27a as a key regulator of metabolic reprogramming and enhancing drug resistance in CRC cells. The study revealed that miR-27a modulates several tumour-associated pathways that link metabolic rewiring with chemoresistance in CRC. It was found that miR-27a negatively regulates AMPK and positively regulates mTOR pathway to force anaerobic glycolytic metabolism supporting tumour growth and chemoresistance (Barisciano et al., 2020). Interestingly, a recent study has shown the colorectal tumour-derived exosomes could activate hepatic stellate cells in the liver to enhance lactate metabolism of tumour cells *via* the IL-6/STAT3 pathway to confer the resistance of SN38 (active metabolite of irinotecan) (Li et al., 2020a; Li et al., 2020b). Hence, this indicates a novel mechanism in which the tumour-derived exosomes are involved in regulating the metabolic reprogramming between the tumour cells and the microenvironment to promote drug resistance (Li et al., 2020a; Li et al., 2020b). Previous studies suggest that hypoxia-induced metabolic reprogramming of CRC can be reversed by targeting valine catabolism and the inhibition of PTEN/AKT/HIF1α signalling pathway to interfere with energy production in CRC (Wang et al., 2018; Shan et al., 2019). Alternative strategies to tackle metabolic heterogeneity in CRC warrant further investigation, given that the signalling mechanisms that contribute to metabolic reprogramming in CRC are complex.

Given the increasing complexity of molecular networks in multidrug-resistant CRC, multi-omics approaches encompassing genomics, epigenomics, transcriptomics, proteomics and metabolomics are applied to facilitate cancer biomarker discovery and guide cancer treatment strategies (Tong et al., 2016; Satoh et al., 2017; Ressa et al., 2018; Ishaque et al., 2018). Integrated multi-omics analyses of CRC cell lines have reported genetic and epigenetic alterations in the molecular landscape for CRC carcinogenesis such as (i) the identification of *BRCA1*-centred gene-miRNA-protein regulatory network as the main driver for liver metastasis of CRC and chemoresistance (Gerovska et al., 2020), (ii) co-occurrence of genetic alteration events in CRC that affects drug response (Zhou et al., 2020), (iii) heterogeneous Wnt/β-catenin activity that supports Runt-related transcription factor 2 (RUNX2)-based epigenetic regulation of EMT as the molecular implication for poor survival of CRC patients and failure of anti-cancer therapy (Yi et al., 2020), and (iv) the characterisation of CRC patients’ drug response patterns based on differential DNA methylation profiles in CRC stem cell populations (Visone et al., 2019). To better understand the relationship between immune landscape in the TME and drug efficacy against CRC, computational methods such as (i) the development of artificial intelligence platform to predict immunological responses to ICB therapy in MSI-H tumour (Cao et al., 2020) and (ii) the reconstruction of the intercellular network according to consensus molecular subtypes of CRC (Lee et al., 2020), have enabled the selection of better treatment options for individuals resistant to immunotherapy. Comprehensive analysis of multi-omics data such as metagenomic and metabolomic has also reported the role of gut microbiota in CRC progression and their influences on the DNA methylome of CRC, implying that the metabolic output of gut microbiota and the host’s epigenetic signatures could be important diagnostic targets for CRC management (Yachida et al., 2019; Sobhani et al., 2019; Zouggar, Haebe & Benoit, 2020).

Cancer biopsy-based bulk analysis of CRC is gradually replaced by patient-derived cancer organoid (PDCO) to study therapy resistance *in vitro*. This is because PDCO can recapitulate tumour heterogeneity in the patient tumour which potentiates the study of CRC at the single-cell level (Jeppesen et al., 2017; Chen et al., 2018c; Pasch et al., 2019; Demmers et al., 2020). However, limitations such as the time and cost to grow the organoids as well as limited amount of organoids available prompts the shift of focus to cancer tissue-originated spheroid (CTOS) as an alternative method for measuring chemotherapeutic heterogeneity and high-throughput drug screening for CRC patients (Jeppesen et al., 2017; Kondo et al., 2019). Technical advancement to improve the study of tumour heterogeneity in the spatial context is exemplified by RNA-based *in situ* hybridisation which complements the current genetic method to ease the detection of rare subclones in CRC (Baker et al., 2017). Non-invasive methods such as comprehensive genotyping of circulating tumour DNA (ctDNA) and integrated multi-omics data analyses for gene alteration events relating to drug responses, are also useful in identifying druggable mutational targets for personalising cancer medicine (Cao et al., 2019; Zhou et al., 2020). Besides improving on experimental methods to better understand tumour heterogeneity in CRC, the application of modern bioinformatic practice (*e.g.*, reference component analysis of single-cell transcriptomes) and machine learning algorithm (*e.g.*, deep learning for the prediction of treatment efficacy on CRC patients) are equally important to the management of increasingly complex cancer research data which influences cancer treatment policy (Li et al., 2017; Skrede et al., 2020; Vera-Yunca et al., 2020).

## Conclusions

Therapy resistance in CRC remains a major obstacle to CRC management due to alterations in the molecular landscape that drive the survival of cancer cells. In particular, dysregulation of MAPK pathway, PI3K/PKB pathway, Wnt/β-catenin pathway and Notch pathway are frequently reported to induce resistance to anti-cancer drugs targeting CRC cells. On the other hand, other signal transduction pathways such as TGFβ/Smad, BMP and Hedgehog pathways have also been implicated in the development of therapeutic resistance in CRC but are not well studied yet. The complexity of drug resistance mechanisms is further widened by pre-existing genetic heterogeneity in CRC and cellular components of the TME (*e.g.*, stromal cells, immune cells and gut bacteria) which results in the evolution of drug-resistant tumour subclones. To address this problem, integrated multi-omics data analysis using modern computational methods, three-dimensional cell culture model and other robust experimental methods are needed to identify new cancer biomarkers and drug targets for CRC treatment. Despite the effort in combating multidrug-resistant CRC, further studies are warranted to generate quality results for better cancer care delivery.

## Disclaimer

Most experimental findings discussed this review are derived from studies using laboratory-based cancer cell lines. Hence, the results should be interpreted with caution and be further validated in animal models and human clinical studies.

## Additional Information and Declarations

### Data Availability

The following information was supplied regarding data availability: There are no raw data
